# Maize and heat stress: Physiological, genetic, and molecular insights

**DOI:** 10.1002/tpg2.20378

**Published:** 2023-08-16

**Authors:** Ivica Djalovic, Sayanta Kundu, Rajeev Nayan Bahuguna, Ashwani Pareek, Ali Raza, Sneh L. Singla‐Pareek, P. V. Vara Prasad, Rajeev K. Varshney

**Affiliations:** ^1^ Institute of Field and Vegetable Crops National Institute of the Republic of Serbia Novi Sad Serbia; ^2^ National Agri‐Food Biotechnology Institute Mohali India; ^3^ Stress Physiology and Molecular Biology Laboratory, School of Life Sciences Jawaharlal Nehru University New Delhi India; ^4^ Fujian Provincial Key Laboratory of Crop Molecular and Cell Biology, Oil Crops Research Institute, Center of Legume Crop Genetics and Systems Biology/College of Agriculture Fujian Agriculture and Forestry University (FAFU) Fuzhou Fujian China; ^5^ Plant Stress Biology International Centre for Genetic Engineering and Biotechnology New Delhi India; ^6^ Feed the Future Innovation Lab for Collaborative Research on Sustainable Intensification Kansas State University Manhattan KS USA; ^7^ State Agricultural Biotechnology Centre, Centre for Crop and Food Innovation, Food Futures Institute Murdoch University Murdoch Western Australia Australia

## Abstract

Global mean temperature is increasing at a rapid pace due to the rapid emission of greenhouse gases majorly from anthropogenic practices and predicted to rise up to 1.5°C above the pre‐industrial level by the year 2050. The warming climate is affecting global crop production by altering biochemical, physiological, and metabolic processes resulting in poor growth, development, and reduced yield. Maize is susceptible to heat stress, particularly at the reproductive and early grain filling stages. Interestingly, heat stress impact on crops is closely regulated by associated environmental covariables such as humidity, vapor pressure deficit, soil moisture content, and solar radiation. Therefore, heat stress tolerance is considered as a complex trait, which requires multiple levels of regulations in plants. Exploring genetic diversity from landraces and wild accessions of maize is a promising approach to identify novel donors, traits, quantitative trait loci (QTLs), and genes, which can be introgressed into the elite cultivars. Indeed, genome wide association studies (GWAS) for mining of potential QTL(s) and dominant gene(s) is a major route of crop improvement. Conversely, mutation breeding is being utilized for generating variation in existing populations with narrow genetic background. Besides breeding approaches, augmented production of heat shock factors (HSFs) and heat shock proteins (HSPs) have been reported in transgenic maize to provide heat stress tolerance. Recent advancements in molecular techniques including clustered regularly interspaced short palindromic repeats (CRISPR) would expedite the process for developing thermotolerant maize genotypes.

## Core Ideas

- Maize is an important food, nutrition, and climate security crop around the world.
- Heat stress negatively impacts the growth, development, and yield of maize.
- Genotypes respond to heat stress at physiological, biochemical, and molecular levels.
- Current knowledge and advances in traits and genotypic responses are discussed.

**Abbreviations**
CT: canopy temperatures
CRISPR: clustered regularly interspaced short palindromic repeats
GWAS: genome wide association studies
HNT: heat stress at night time
HSFs: heat shock factors
HSPs: heat shock proteins
LD: linkage disequilibrium
QTLs: quantitative trait loci
ROS: reactive oxygen species
SNPs: single nucleotide polymorphism
TALENs: transcription activator‐like effector nucleases
VPD: vapor pressure deficit
ZFNs: zinc‐finger nucleases

## 1 INTRODUCTION

**FIGURE 1:**
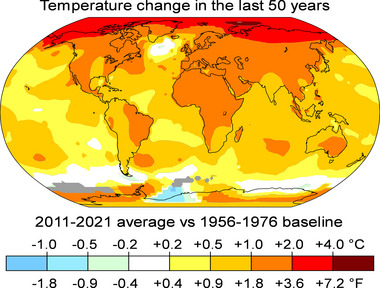
The average surface air temperature changes between 2011 to 2012 compared with the 1965 to 1976 average. *Source*: NASA (https://data.giss.nasa.gov/gistemp/).

Adverse influences of global climate change factors on crop yields and production are major risks to food and nutrition security in the present century. Hence, adaptation and mitigation of adverse impacts of climate are crucial to sustaining the food supply for the increasing world population (Farooq et al., 2022; Rivero et al., 2022). The effect of climate change and climate variability on agricultural productivity will be the most significant in the tropics, sub‐tropics, and semi‐arid tropics of Africa and South Asia. These regions are estimated to be especially susceptible to various stresses and low adaptive capability (Aryal et al., 2020; Boko et al., 2007). Current climate change is negatively impacting crop production globally (Cairns et al., 2013; Farooq et al., 2022), particularly in the foremost staple food crops, such as maize (*Zea mays* L.), rice (*Oryza sativa* L.), and wheat (*Triticum aestivum* L.). Several environmental and edaphic factors such as rising mean temperature, regional and extreme heat waves, drought spells, expanding soil salinity, nutrient imbalance, and accumulation of toxic heavy metals in soil are associated with climate change (Dhankher & Foyer, 2018; Rivero et al., 2022). However, the rising mean temperature is a major climatic factor (Figure 1), which is documented to reduce crop production worldwide (Haider et al., 2022; Raza et al., 2021; Raza et al., 2023; Sharma et al., 2022). Reports from NASA's Goddard Institute of Space Studies (GISTEMP, 2023) stated that global mean temperature has increased by 1.1°C since 1880, while heat waves have become more prevalent in past decades. Consequently, the increase in mean day/night temperature and occurrences of heat waves at the regional scale have been becoming a threat to crop production particularly in rainfed agriculture systems (Chakraborty et al., 2019). A sudden or gradual rise in temperature beyond the threshold that could trigger irreparable damage to plant growth and development is defined as heat stress (Bahuguna & Jagadish, 2015). Numerous climate modeling studies suggested that heat stress events will be more frequent in the future environment, which may signify a major constraint to crop productivity and global food security (Masson‐Delmotte et al., 2021; Rivero et al., 2022; Senguttuvel et al., 2022).

Maize is the most widely cultivated C4 plant covering 197 M ha of land, globally and the third most important cereal crop contributing to global food security (FAOStat, 2021). The U.S. Department of Agriculture (USDA) reported that more than 1150 million tons of maize has been produced worldwide in 2022/2023 market year. However, maize is experiencing frequent and severe heat stress (Wang et al., 2021a) impacting the functioning of physiological processes including changes at morphological, physiological, biochemical, and molecular levels, which ultimately impacts growth and yield. Evidences are accumulating on the adverse impact of heat waves on maize production across Europe (Hawkins et al., 2013), the United States (Lobell & Asner, 2003), sub‐Saharan Africa (Lobell et al., 2011), and China (Gao et al., 2021; Tao et al., 2016). Therefore, plant biologists, breeders, and crop scientists are prioritizing the focus on improving the heat tolerance in maize (Iqbal et al., 2020; Rahman et al., 2015). In the recent past, several crop growth models have been extensively utilized to assess the adverse consequences of heat stress on maize production (Gabaldón‐Leal et al., 2016; Rezaei et al., 2015). Nevertheless, these models have not been explicitly calibrated or do not have mechanistic attributes to quantify extreme heat stress events. Consequently, these simplified models might have under‐projected and over‐projected the influences of heat stress on maize production. For instance, heat stress during the flowering phase might trigger a significant yield decline, however, similar temperature stress at the vegetative phase may not cause similar impact on yield (Lizaso et al., 2018; Wang et al., 2021b). For example, heat stress did not affect maize growth and grain yield when occurred prior to the ninth leaf phase (Lizaso et al., 2018). Notably, recent investigations have described the critical phases in maize that explain yield losses due to heat stress. Both short and long duration heat stress is known to negatively impact reproductive processes and harvest index of cereals (Prasad et al., 2017). Tassel and ear formation occur at the six visible leaf collar stages (V6 stage), which is one of the critical stages susceptible to severe stress. Hence, heat stress particularly during the V6 stage may cause the formation of underdeveloped tassels, which eventually die due to desiccation, known as tassel blast (TSBL). Further, reproductive tissue injury in the form of TSBL has been reported to reduce viable pollen numbers (McNellie et al., 2018). Apart from pollen formation, pollen development is also extremely susceptible to heat stress. Moreover, damaged pollen tube formation along with impaired stigma receptivity due to silk parchedness could directly impact the seed set, that eventually results in lower yield under heat stress (Lohani et al., 2020; Zinn et al., 2010). Conversely, ear shoots advancement is necessary for silking and grain filling, thus, heat stress at the pre‐anthesis stage could be detrimental to yield in maize (Suwa et al., 2010). On the other hand, heat stress during the grain filling period augments the respiratory carbon losses and impairs the source to sink assimilate partitioning (Yang et al., 2018). In addition, it also shortens the active grain filling period (Yang et al., 2017), eventually leading to the reduction in kernel weight and deterioration in kernel quality (Chukwudi et al., 2021; Yang et al., 2018). Therefore, it is crucial to better understand the molecular, biochemical, and physiological processes across different growth phases, particularly during the reproductive and grain filling stages, which eventually impair maize growth, development, and yield in maize under heat stress conditions. This review provides an overview of the impacts of heat stress on maize crop. Further, it highlights the importance of stress‐related trait‐based phenotyping and important molecular mechanisms helping in identifying new sources for heat stress tolerance and opportunities to improve heat tolerance in maize.

## 2 HEAT STRESS IN NATURAL ENVIRONMENTS

Technical advances in cultivation practices and expansion of the area for maize cultivation (doubled up from 106 M ha in 1963 to currently 197 M ha globally) have increased maize production approximately by five fold (Erenstein et al., 2022). Nevertheless, it has been estimated that maize yield would reduce by 7.4% for every 1°C increase in temperature (Zhao et al., 2017). The optimum temperature for maize cultivation is 28 to 32°C, which is relatively higher than the optimum temperatures required for the production of other cereal crops (Arnold, 1974). However, a direct effect of heat stress on photosynthesis and vegetative growth has been documented when the temperature exceeds 38°C (Crafts‐Brandner & Salvucci, 2002). On the other hand, a prolonged exposure to temperature above 35°C, specifically during the reproductive stage is detrimental to reproductive success and seed set (Shao et al., 2021), leading to reproductive biomass and yield loss (Carter et al., 2016). During the reproductive stage, male flowers or tassels are the most vulnerable tissue to heat stress than female counterparts (Dong et al., 2021). Thus, heat stress results in inhibition of anther dehiscence and reduction in pollen viability, pollen germination, and pollen tube growth. Expanded anthesis–silking interval (ASI) due to delayed silking, parched silk, and suppression in fertilization lead to kernel abortion lowering kernel number per cob (Bakhtavar et al., 2015; Carberry et al., 1989; Hatfield & Prueger, 2015; Li & Howell, 2021). Heat stress after pollination also results in kernel abortion due to reduction in carbohydrate availability and damage in carbon metabolism (Niu et al., 2021). Conversely, rise in night temperature (heat stress at night time [HNT]) during silking could affect the kernel setting by increased rate of development, which inhibits photo‐assimilate production and availability to the kernel (Cantarero et al., 1999). HNT particularly during the grain filling stage has been reported to reduce grain weight (Cantarero et al., 1999). Moreover, HNT leads to an increase in night respiration causing rapid degradation of carbohydrates. In addition, HNT interferes in source to sink carbon dynamics by affecting non‐structural carbohydrate metabolizing enzymes such as soluble starch synthase and starch branching enzymes (Kettler et al., 2022; Yang et al., 2018).

**Core Ideas**

- Maize is an important food, nutrition, and climate security crop around the world.
- Heat stress negatively impacts the growth, development, and yield of maize.
- Genotypes respond to heat stress at physiological, biochemical, and molecular levels.
- Current knowledge and advances in traits and genotypic responses are discussed.

While the detrimental effect of heat stress on maize is well documented, the impact and severity of heat stress cannot be explained without including some covariables such as humidity, vapor pressure deficit (VPD), soil moisture deficit, and solar radiation (Carter et al., 2016; Kebede et al., 2012; Thayil et al., 2020) that interact with ambient temperature, and the cumulative effect of these factors affect growth, flowering, phenophase transition, and seed set in plants (Bahuguna & Jagadish, 2015). Carter et al. (2016) reported that tropical maize plants grown under irrigated condition showed no yield reduction under heat stress, whereas yield of rainfed maize was significantly reduced due to heat stress. Authors further demonstrated that heat stress (32–34°C) had little impact on maize production under irrigated condition, and soil moisture deficit predominantly determined the severity of heat stress (Carter et al., 2016). Besides soil moisture content, air moisture and VPD are closely associated with temperature impact on plants. VPD is a function of maximum temperature (*T*_m_) and relative humidity under maximum temperature (Thayil et al., 2020). Low VPD along with high temperature stress has been reported to cause substantial yield loss in maize due to low transpiration from stomata limiting effective evaporative cooling from plant surface. On the other hand, the atmosphere becomes more moisture demanding under high VPD, which results in higher water loss from plant surface and causes physiological drought (Seetharam et al., 2021). Experiments on spring maize grown under heat stress (>37°C) in multiple locations, with the humid and dry condition in South Asia showed the differential impact of heat stress across the environmental conditions showing the interaction of temperature and air moisture. Interestingly, the crops, grown under warm–dry condition showed higher grain yield loss as compared to warm–humid condition plausibly due to the pronounced effect of soil moisture deficit condition (Thayil et al., 2020; Vinayan et al., 2019). On the other hand, frequent irrigation under drought could maintain tissue hydration and transpirational cooling resulting in lower grain yield loss (Siebert et al., 2014). Hence, crop response to heat stress may vary substantially with changes in moisture levels in the air and soil. Canopy temperatures (CT) is very often used as a surrogate method to predict the level of soil moisture deficit condition (González‐Dugo et al., 2006; Reynolds et al., 2007). Interestingly, maize plants could experience severe heat stress both under high VPD and soil moisture deficit condition, as in both cases evapotranspiration is not able to meet the moisture demand of the atmosphere, and it increases canopy and tissue temperatures (Kebede et al., 2012). Besides relative humidity, soil moisture, and VPD, solar radiation reported to determine the impact of heat stress as it may elevate the level of heat stress severity by enhanced photoinhibition and tissue temperature (Kebede et al., 2012). On the other hand, an increase in soil temperature causes rapid weathering of mineral clays such as kaolinite, and that results in low retention of nutrients and loss of important nutrients like Ca and Mg. It also affects plants’ water uptake as the rate of evaporation from soil increases. These heat interacting factors vary at regional scale. Thus, the interaction between growing genotypes and prevailing environmental factors in a particular location determines the crop phenotype (Thayil et al., 2020). Besides interaction with other abiotic factors, heat stress has been documented to increase plants’ susceptibility toward pathogen invasion by interfering with plant defense mechanisms. For example, heat stress affects the synthesis of plant defense enzymes, salicylic acid which elicits the plant immune system (Cohen & Leach, 2020). Moreover, Duke and Doehlert demonstrated that heat stress restricted pathogenesis‐related protein and enzyme synthesis in maize kernel, and inhibited kernel's defensive ability to defeat the pathogen invasion. For instance, high temperature accompanied by prolonged dry period has been reported to cause *Aspergillus* ear rot in maize (Barošević et al., 2022). Although it is important to study plants’ response to pathogen attack under warming climate, no studies exist on maize under natural environment.

## 3 PHYSIOLOGICAL RESPONSES AND TRAIT‐BASED PHENOTYPING

**FIGURE 2:**
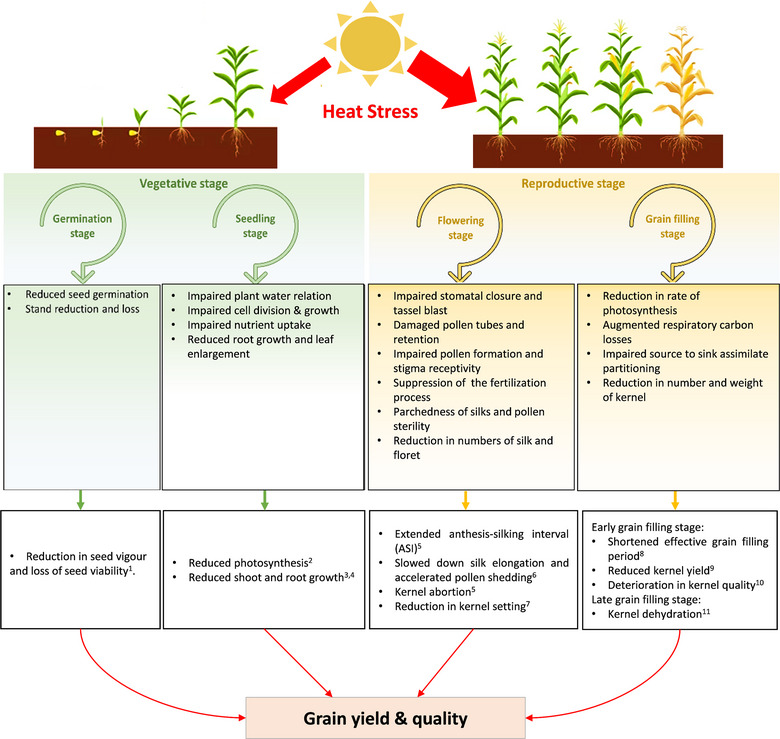
An overview of the impact of heat stress at different developmental stages of maize, impairing maize growth, production, and quality. The reproductive and grain filling period are the most susceptible stages in maize under heat stress. The red colored arrows depict the adverse effect of heat stress and the thickness of those arrows shows the degree of impact on these stages (Chukwudi et al., 2021; Karim et al., 2000; Lizaso et al., 2018;Mathur et al., 2014; Niu et al., 2021; Omoarelojie et al., 2020; Shao et al., 2021; Teng et al., 2022; Wang et al., 2021b; Yang et al., 2017, 2018).

Exposure to heat stress severely affects physiological processes such as seed germination and vigor, root growth, leaf enlargement, reproductive development, ASI, photosynthesis, grain filling duration, and rate of grain filling, which eventually reduce yield and grain quality (Figure 2). At the cellular level, the rapid production of reactive oxygen species (ROS) leads to cellular membrane damage (CMD), protein degradation, and inhibition of chlorophyll synthesis (Tas, 2022). Heat stress also interferes with gaseous exchange by altering stomatal conductance, which leads to decrease in tissue water potential of plants (Urban et al., 2017). Coşkun et al. (2011) tested 10 maize inbreds for heat stress tolerance and studied several physiological traits such as leaf temperature (LT), relative injury (RI), and chlorophyll a/b content to evaluate their performance under heat stress. Authors described that the exposure of maize plants to heat stress resulted in increase in leaf temperature (from 36.6 to 39.2°C), and a decrease in cellular membrane integrity. Further, an increase in RI caused inhibition of chlorophyll synthesis and rapid senescence, which eventually resulted in insufficient accumulation of photo‐assimilates into the grains. Conversely, a negative correlation was observed between CMD and leaf water potential (LWP). An increase in CMD caused decrease in LWP, and inhibition of chlorophyll synthesis as well as degradation of chlorophyll a/b. Moreover, total phenol content (TPC) was also found to be decreased in susceptible genotypes under heat stress (Tas, 2022).

Environment‐specific trait‐based phenotyping has been suggested as the best way to introduce heat stress tolerance in plants (Hall, 2011). Several traits in maize have been reported that can be targeted for heat stress avoidance and/or tolerance. However, trait selection particularly at the flowering, and grain filling stage could be the most effective as these are the most sensitive stages of maize under heat stress (Commuri & Jones, 2001; Karim et al., 1999; Thayil et al., 2020). Leaf area, leaf elongation rate, and photosynthetic rate have been suggested as the potential traits under heat stress in maize during the early vegetative or seedling stage (Karim et al., 1999). Nelimor et al. (2019) described 13 traits that are directly or indirectly associated with grain yield and distributed these traits in three groups according to their order of effectiveness under heat stress. The first order comprises of traits such as ear aspect, plant aspect, and root lodging, where ear aspects had the strongest effect on grain yield under heat stress. The second‐order traits included ears per plant, husk cover, plant height, and days to 50% anthesis, which were indirectly related to grain yield. However, both ears per plant and husk cover had a strong effect on both the ear aspects and plant aspects. The third‐order traits were described as days to 50% silking, ASI, ear rot, stay green characteristics, TSBL and ear height, and it was observed that ASI and ear rot had a strong indirect association with grain yield. Similarly, Chen et al. (2012) conducted phenotypic analysis of 11 inbred lines of maize under heat stress (∼36°C). The phenotypic traits targeted for the study were leaf firing, leaf blotching, and tassel blasting. Authors observed that leaf firing and leaf blotching were the most noticeable heat sensitive phenotypes during the vegetative stage. Senescence, kernel per row, tassel sterility, pollen viability, and stigma receptivity have also been documented as potential traits that could be utilized to characterize thermotolerance in maize (Alam et al., 2017; Noor et al., 2019).

Traits identification using natural genetic diversity or mutant population is promising. However, precise phenotyping of a diverse population has been a major bottleneck for crop improvement programs (Feng et al., 2017). Recent advancements in the field of phenomics, and the development of high‐throughput phenotyping platforms made it possible to screen and characterize large germplasm in a very short period of time, which has tremendously expedited the varietal development (Song et al., 2021). Moreover, the inclusion of artificial intelligence and robotics in phenotyping has improved precision and reliability (Irshat et al., 2018; Pound et al., 2017). Nevertheless, heat stress tolerance is a complex trait with genotype by environment interaction effect, which requires trait plasticity and stability and must be considered while characterizing genotypic response under specific environment.

## 4 EXPLORING GENETIC VARIATION FOR HEAT STRESS TOLERANCE

Plant response to heat stress is a complex process involving multiple components at the cellular level and polygenic traits at the whole plant level. Indeed, a single exposure to heat stress has been reported to induce more than 5000 genes in maize plants (Joshi et al., 2021). Traits contributing to heat stress tolerance such as pollen viability and ASI are governed by multiple genes/loci (Frova & Sari‐Gorla, 1994; Parrado et al., 2021). Thus, mining of heat stress tolerance‐associated QTLs/genes is crucial to develop heat tolerant high‐yielding cultivars (Inghelandt et al., 2019; McNellie et al., 2018). Recent advancements in genomics, marker‐assisted selection, and phenomics allowed the identification of QTLs and genes through genome wide association studies (GWAS), and advanced mapping populations. A number of QTLs have been reported in maize, which are related to heat stress tolerance (Table 1), and a number of genes and transcription factors induced under heat stress were reviewed by El‐Sappah et al. (2022) that can be used in precise genetic engineering and breeding for heat stress tolerance.

**TABLE 1:** Some of the QTLs/genes reported in maize having role in heat stress tolerance are discussed along with their chromosomal location, position, growth stage of expression, and traits governed.

Sl. No.	QTLs/genes identified	Chromosome no.	Position	Growth stage	Trait/mechanism	Reference
1.	*Zm00001d043634* *Zm00001d025343*	3	–	Vegetative stage	Plant death (PD) and Leaf firing (LF)	(Reddy et al., 2017; Tůmová et al., 2018)
2.	Q_HSI:LS_ (*GRMZM2G094990*)	9	64		HSI_LS_	(Frey et al., 2016)
3.	QTL_EVS_LF_NC350_	9	46		EVS_LF_NC350	(McNellie et al., 2018)
		10	45			
4.	QTL_EVS_LB_	1	74		EVS_LB	(McNellie et al., 2018)
		10	43			
5.	QTL_MVS_LF_NC350_	5	44		MVS_LF_NC350	(McNellie et al., 2018)
		9	57			
		10	40			
6.	QTL_MVS_LB_ (B73 × NC350)	1	77		MVS_LB	(McNellie et al., 2018)
7.	QTL_MVS_LB_ (B73 × CML103)	2	123			
8.	QTL_LVS_LF_NC350_	5	43		LVS_LF_NC350	(McNellie et al., 2018)
		9	52			
		10	40			
9.	QTL_LVS_LF_B73_ (B73 × NC350)	1	60		LVS_LF_B73	(McNellie et al., 2018)
10	QTL_LVS_LB_ (B73 × NC350)	8	91		LVS_LB	(McNellie et al., 2018)
		10	38			
11.	QTL_LVS_TSBL_	5	114		LVS_TSBL	(McNellie et al., 2018)
12.	QTL_LVS_LF_CML103_	2	113		LVS_LF_CML103	(McNellie et al., 2018)
		3	107			
13.	QTL_LVS_LF_B73_ (B73 × CML103)	1	63		LVS_LF_B73	(McNellie et al., 2018)
14.	QTL_LVS_LB_ (B73 × CML103)	2	123		LVS_LB	(McNellie et al., 2018)
		3	134			
15.	QTL_LVS_PD_	3	106		LVS_PD	(McNellie et al., 2018)
16.	Q_HSI:LLa_	2	37		HSI_LL_	(Inghelandt et al., 2019)
17.	Q_HSI:LLb_	10	30			
18.	Q_HSI:PH_	9	3		HSI_PH_	(Inghelandt et al., 2019)
19.	Q_HSI:SC_	9	74		HSI_SC_	(Inghelandt et al., 2019)
20.	Q_HSI:SD_	5	101		HSI_SD_	(Inghelandt et al., 2019)
21.	Q_HSI:LR_	2	82		HSI_LR_	(Inghelandt et al., 2019)
22.	*Os*MYB55	–	–		–	(Casaretto et al., 2016)
23.	*Zm*DREB2A	–	–	Vegetative and reproductive stages	–	(Qin et al., 2007)
24.	Q_HSI:FF_ *(GRMZM2G148998)*	2	15	Flowering	HSI_FF_	(Frey et al., 2016)
25.	Q_HSI:MFa_ *(GRMZM2G148998)*	2	15		HSI_MF_	(Frey et al., 2016)
26.	Q_HSI:MFb_ *(GRMZM2G436710*)	5	140		HSI_MF_	(Frey et al., 2016)
27.	Q_HSI:MFc_	9	13		HSI_MF_	(Frey et al., 2016)
28.	Q_HSI:DYa_ (*GRMZM2G115658, GRMZM2G537291*)	2	45	Grain filling stage	HSI_DY_	(Frey et al., 2016)
29.	Q_HSI:DYb_ (*GRMZM2G324886*)	3	131		HSI_DY_	(Frey et al., 2016)
30.	Q_HSI:DYAa_	2	45		HSI_DYA_	(Frey et al., 2016)
31.	Q_HSI:DYAb_ *(GRMZM2G324886*)	3	131		HSI_DYA_	(Frey et al., 2016)
32.	*pgd1 and pgd2*	–	–		–	(Ribeiro et al., 2020.)
33.	*PSY1*	6	–		–	(Li et al., 2008)

Abbreviations: DY, dry yield; DYA, adjusted dry yield; EVS, early vegetative stage; FF, female flowering; HSI, heat susceptibility index; LB, leaf blotching; LF, leaf firing; LL, leaf length; LR, leaf growth rate; LS, leaf scorching; LVS, late vegetative stage; MF, male flowering; MVS, middle vegetative stage; OsMYB55, *Oryza sativa* myeloblastosis 55; *pgd*, 6‐phosphogluconate dehydrogenase; PH, plant height; *PSY1*, phytoene synthase; SC, leaf scorching; SD, leaf greenness; TSBL, tassel blast; PD, plant death, *Zm*DREB2A, *Zea mays* dehydration responsive element binding protein 2A.

Genetic variation in potential traits is important for breeding programs and the development of mapping population (Nawab et al., 2008). The elite maize cultivars have limited genetic variation particularly for heat stress tolerance (Nelimor et al., 2019). Hence, exploitation of existing natural resources in landraces and wild accessions of maize crop hold promise to identify potential heat responsive traits (Nelimor et al., 2019). However, the inclusion of landraces into thermotolerance breeding programs in maize would require some intermediate steps, such as targeted selection of landrace(s), adequate variation for the desired trait(s), and development of a core collection of accessions (Würschum et al., 2022). In addition, landrace(s) needs to be top crossed to check the combining ability with the contrasting gene pool, and depending on the number of targeted traits and variation present, single or multiple landraces can be selected for crop improvement programs (Würschum et al., 2022). Ultimately, homozygous lines per landrace could be developed for association mapping through a significant association study between markers and trait(s) to detect major QTLs responsible for the expression of quantitative traits, for example, heat stress tolerance (Yuan et al., 2019). The efficiency of association mapping depends on the size of the mapping population and its resolution, which totally depends on the linkage disequilibrium (LD) structure of the QTL (Würschum et al., 2022). LD decays faster in landraces than in elite cultivars. Interestingly, the rate of LD decay was found to be even faster in a diverse collection of maize genotypes as compared to the landraces, however, their adaptability to adverse environment was poor (Würschum et al., 2022). Thus, promising maize landrace(s) could be used in genome wide association mapping of thermotolerance targeted trait(s), and to identify potential QTLs with large effect size and further, the dominant gene(s) responsible for the trait(s) (Ahmed et al., 2022; Nelimor et al., 2019). Single nucleotide polymorphism (SNP) markers are mostly used as markers in association studies (Sivakumar et al., 2019). Ahmed et al. (2022) sequenced 275 maize inbred lines, grown under two different temperature regimes including 35 and 45°C, and identified 1,70,098 SNPs. Subsequently, genome wide association of trait such as average pollen germination percentage with identified SNPs revealed that each line had 4–10, 3–13, and 5–13 beneficial alleles for pollen germination percentage at 35°C, pollen germination percentage at 45°C, and pollen germination percentage ratio. These alleles can be used for developing improved tropical maize varieties for heat stress tolerance.

A population of 45 recombinant inbred lines (RILs) were studied in maize for mining of QTLs, particularly for two traits such as injury of pollen grain germinability and injury of pollen tube growth under heat stress. Restriction fragment length polymorphism (RFLP) markers were used for genome wide association mapping, and a total of 11 QTLs were identified (5 for injury of pollen grain germinability and 6 for injury of pollen tube growth). Chromosomal locations of these identified QTLs indicated that there was no correlation between the genes governing injury of pollen grain germinability and injury of pollen tube growth. It was also described that QTLs of injury of pollen tube growth and membrane stability, a trait that is important for maintaining membrane fluidity under heat stress, share a common region on chromosome number 8. Moreover, no significant correlation was found between injury of pollen tube growth and pollen grain germination under non‐stressed condition. These findings revealed that QTLs for injury of pollen grain germinability played a role in thermotolerance whereas, QTLs for injury of pollen tube growth were found to be less functional under high temperature stress (Frova & Sari‐Gorla, 1994). Similarly, two RIL populations, B73 × NC350 (185 RILs) and B73 × CML103 (195 RILs) of maize were studied for foliar and tassel related heat tolerance traits (McNellie et al., 2018). Authors reported 22 QTLs on different chromosomes, which were associated with traits such as leaf firing (LF), leaf blotching (LB), TSBL, and plant death (PD). In the B73 × NC350 population, 15 QTLs were identified on chromosome nos. 1, 5, 8, 9, and 10 under eight heat stress traits during early, mid, and late vegetative stages. Authors also reported that QTLs for traits, LF_NC350 and LB were co‐localized on chromosome no. 10, and the tolerant allele was donated by the parent, B73. On the other hand, 7 QTLs were mapped on chromosome nos. 1, 2, and 3 under five traits in the B73 × CML103 population. The QTLs for traits, LF_CML103 and LVS_PD were mapped 1 cM (centi‐Morgan) apart on chromosome no. 3, implying that the QTLs captured the same locus for heat tolerance both in leaf and tassel. Frey et al. (2016) studied intra‐ and inter‐pool dent and flint corn population under varying environmental conditions. Authors observed that dent corn was more tolerant to heat stress, particularly at the reproductive stages, and identified 11 potential QTLs related to heat stress tolerance. Moreover, 6 heat tolerant and 112 heat responsive candidate genes associated with the QTLs were identified in the study, which were majorly confined to the flowering and grain filling stages.

Another method of generating genetic variation within a population is through mutation breeding, specifically when existing resources have narrow genetic background (Begna, 2021; Ruswandi et al., 2014). The narrow genetic background can be broadened artificially by inducing mutation using physical and chemical mutagens. Many physical mutagens (e.g., gamma ray, x‐ray, and UV rays) are commonly used, but gamma irradiation is widely preferred and used (Çelik & Atak, 2017). On the other hand, ethyl methane sulfonate is mostly used among chemical mutagens (Chaudhary et al., 2019). These mutagens can be used to cause random changes in the genetic materials (Settles, 2020). Mutant populations developed from these seeds could show positive mutations, which could be useful for effective selection. Other than random mutation, specific gene targeted mutation is also effective in developing thermotolerance in maize. For example, point mutation in *Shrunken2* (*Sh2*) gene improved the level of interaction between the subunits of maize endosperm‐specific ADP‐glucose phosphorylase enzyme under heat stress. ADP‐glucose phosphorylase is essential for starch synthesis and its storage into maize kernel, and its activity is affected by heat stress (Greene & Hannah, 1998).

Genes encoding heat shock factors (HSFs) are considered important for developing genetically modified heat tolerant crops. In maize, 31 HSFs including HSFA1, HSF3, HSF4, HSF5, HSF6, HSF23, and HSF25 were identified, which are documented to regulate the expression of different heat shock proteins (HSPs), and contributing to the heat stress tolerance (Lin et al., 2011). Indeed, transgenic maize with potential HSFs show promising results under heat stress. For example, over‐expression of OsMYB55 recorded higher biomass accumulation and reduced leaf damage in transgenic maize during and after heat stress exposure as compared to wildtype (Casaretto et al., 2016). Moreover, over‐expressed OsMYB55 (OsMYB55‐OE) plants also showed tolerance to drought stress (Casaretto et al., 2016). On the other hand, it has been reported that maize plants exposed to higher night temperature experience grain yield loss due to the reduced activity of chloroplast localized enzyme 6‐phosphogluconate dehydrogenase (6PGDH) also known as PGD3, resulting in the poor accumulation of kernel starch (Cantarero et al., 1999). Gene 6PGDH has two other isoforms localized in the cytoplasm, PGD1 and PGD2, but, only PGD3 is reported as heat liable (Spielbauer et al., 2013). Subsequently, Ribeiro et al. (2020) fused chloroplast targeting peptide sequence, *Waxy 1* with *pgd1* and *pgd2*, and found that the fusion proteins, WPGD1 and WPGD2, were complementing PGD3 function resulting in higher grain yield and biomass under high night temperature.

Although, single dominant gene transformation through transgenic approaches did not give very promising results in developing heat stress tolerant genotypes, mainly due to the complexity of the trait (Jha et al., 2021; Noor et al., 2019). However, recent advances in genome editing techniques such as zinc‐finger nucleases (ZFN), transcription activator‐like effector nucleases (TALEN), and clustered regularly interspaced short palindromic repeats (CRISPR‐Cas) have been now deployed to develop abiotic stress tolerant crop varieties (Ainley et al., 2013; Bhat et al., 2021; Char et al., 2015). Development of drought tolerant maize genotypes through targeted insertion of maize GSO2 promoter into the 5ʹ‐untranslated region (5ʹ‐UTR) of *ARGOS8* gene using CRISPR‐Cas9 showed enhanced tolerance to drought stress (Shi et al., 2017). Similarly, Zhu et al. (2016) used type‐II CRISPR‐Cas9 for targeted mutagenesis of maize phytoene synthase gene (*PSY1*), for identifying its role under heat stress. Authors demonstrated that *PSY1* has an important role in thermotolerance in maize as it helps in maintaining the synthesis of leaf carotenoids, which helps in reducing oxidative damage under heat stress.

## 5 MOLECULAR MECHANISM(S)

**FIGURE 3:**
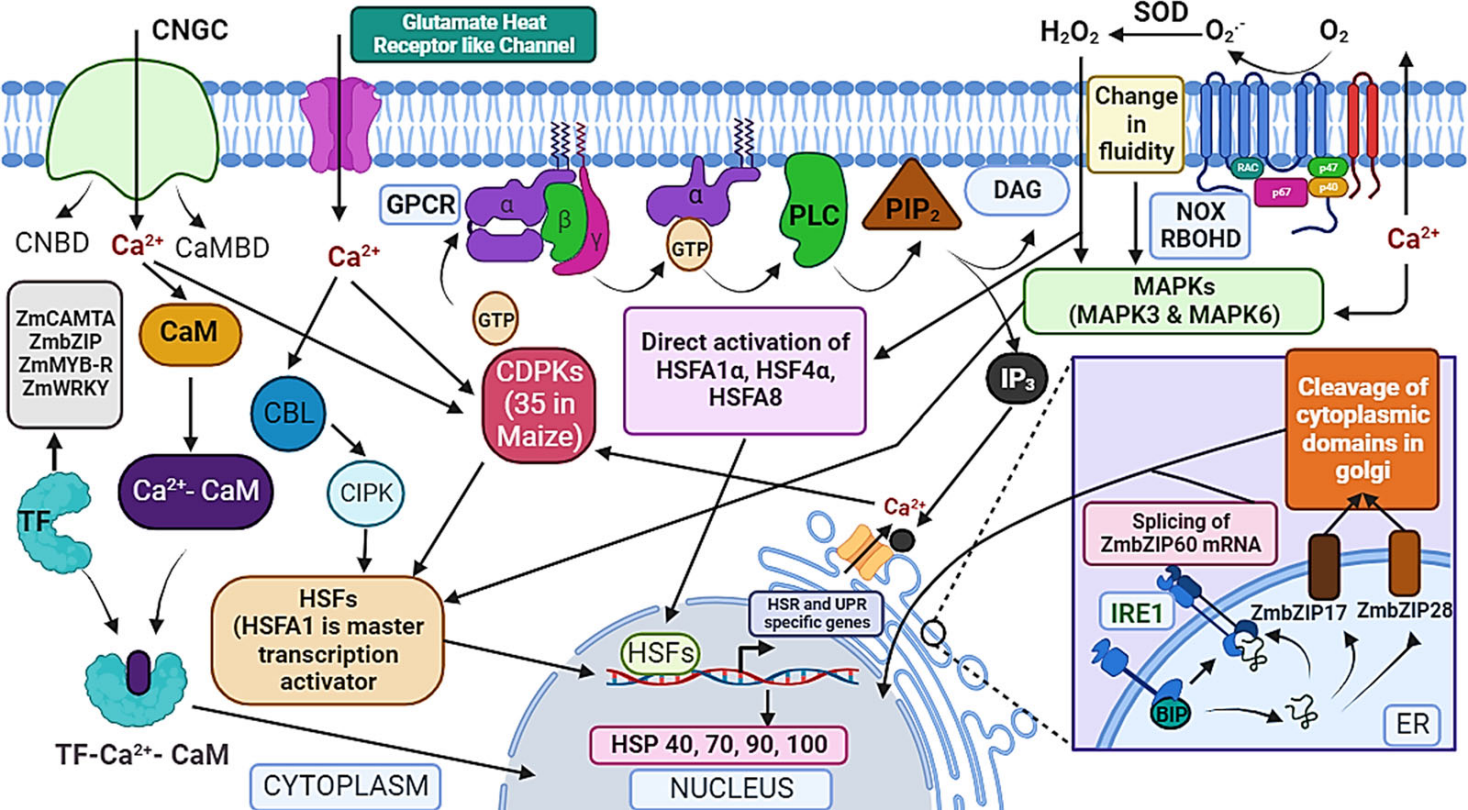
Schematic diagram showing heat stress response in maize at the cellular level. Cellular responses are initiated to acclimatize and protect cells from heat stress impact. The signaling starts with physical change at plasma membrane followed by the rapid influx of Ca^2+^ through voltage‐gated calcium channels, CNGC, and glutamate heat receptor‐like channel into the cytoplasm. The influxed Ca^2+^ in the cytoplasm binds to CaM and forms Ca^2+^‐CaM complex, which helps in activation of TFs such as ZmCAMTA, ZmbZIP, ZmMYB‐R, and ZmWRKY. Eventually, these TFs are imported into the nucleus to activate HSP gene expression. On the other hand, the activation of GPCR by the binding of GTP to the α‐subunit, helps in activation of PLC, which causes hydrolysis of membrane‐bound PIP_2_ and produces DAG and IP_3_. IP_3_ regulates Ca^2+^ release from ER. Ca^2+^ influx in cytosol activates CDPKs (35 in maize), CBL, and CIPK proteins, which are followed by the activation of HSFs (e.g., HSFA1, known as master transcription activator). Besides these signaling routes, a rapid production of ROS takes place after change in plasma membrane fluidity and Ca^2+^ efflux. In ER, misfolded proteins recruit BIP from monomeric IRE1, bind to IRE1, and cause its dimerization. The dimeric IRE1 splices ZmbZIP60 mRNA, which codes for UPR‐specific TF in the nucleus. The other way of activating UPR response is through the cleavage of cytoplasmic globular domain of ER membrane‐bound proteins, that is, ZmbZIP17 and ZmbZIP28 in the golgi complex and these domains help in UPR‐specific gene expression. CaM, calmodulin; CaMBD, calmodulin binding domain; CBL, calcineurin B‐like protein; CDPKs, Ca^2+^‐dependent protein kinases; CIPK, CBL‐interacting protein kinase; CNBD, cyclic nucleotide binding domain; CNGC, cyclic nucleotide‐gated channel; DAG, diacylglycerol; ER, endoplasmic reticulum; GPCR, G‐protein coupled receptor; GTP, guanosine triphosphate; HSFs, heat shock factors; HSP, heat shock proteins; HSR, heat shock response; IP_3,_ inositol 1,4,5‐trisphosphate; IRE1, inositol requiring enzyme‐1; MAPKs, mitogen‐activated protein kinases; NOX RBOHD, NADPH oxidase‐respiratory burst oxygen homolog D; PIP_2_, phosphatidylinositol 4,5‐bisphosphate; PLC, phosphoinositide‐specific phospholipase C; ROS, reactive oxygen species; SOD, superoxide dismutase; TF, transcription factors; UPR, unfolded protein response; ZmCAMTA, *Zea mays* calmodulin binding transcription activator; ZmbZIP, *Zea mays* basic leucine zipper domain; ZmMYB‐R, ZmMYB‐R family genes; ZmWRKY, maize WRKY transcription factor ZmWRKY for drought and heat tolerance.

Plants show cellular level responses to environmental stress such as heat repair the damage occurring to cellular components, and augment the defense response for future stress exposures (Li & Howell, 2021). Responses to heat stress at the cellular level have been categorized into two major types: (i) heat shock response, which takes place majorly in the cytoplasm, (ii) unfolded protein response generated in the endoplasmic reticulum (El‐Sappah et al., 2022). These are the responses that are triggered by the accumulation of misfolded proteins to maintain and protect cellular protein molecules from the adverse effect of environmental changes. Besides, heat shock response and unfolded protein response, heat stress specific responses primarily start at the plasma membrane with a physical change in membrane fluidity, which can instantly open up some voltage‐gated Ca^2+^ ion channels, and ROS production in the chloroplast thylakoid membrane (Bahuguna & Jagadish, 2015; Finka et al., 2012; Horváth et al., 2012, 1998; Yamamoto, 2016). Indeed, an increase in membrane fluidity followed by a Ca^2+^ efflux into cytoplasm is considered as a primary response under heat stress (Finka et al., 2012; Mittler et al., 2012). Calcium signals activate mitogen activated protein kinases (MAPKs), which further activates TFs such as HSFA6b, ABF1, CYD5, and MutS2, specific to heat stress tolerance in maize (Gao et al., 2012). On the other hand, heat stress may be sensed through plasma membrane‐bound receptor proteins. The membrane‐bound proteins for heat stress tolerance are cyclic nucleotide‐gated channels, and glutamate heat receptor‐like channels (Liu et al., 2018). The Ca^2+^‐permeable channels reported in plants are voltage‐dependent Ca^2+^‐permeable channel, voltage‐independent Ca^2+^‐permeable channel, depolarization‐activated Ca^2+^‐permeable channel, and hyperpolarization‐activated Ca^2+^‐permeable channel (Swarbreck et al., 2013). In maize, 11 cyclic nucleotide‐gated channels are reported (Hao & Qiao, 2018), and each one has two binding domains, that is, cyclic nucleotide binding domain and calmodulin (CaM) binding domain in the cytoplasmic region (Zelman et al., 2012). Binding of cyclic nucleotides to the cyclic nucleotide binding domain leads to influx of Ca^2+^, which causes the binding of Ca^2+^ to CaM (Kaplan et al., 2007). Eventually, the Ca^2+^–CaM complex helps in activating some transcription factors (TFs) such as ZmCAMTA, ZmbZIP, ZmMYB, and ZmWRKY‐R, reported in maize. However, ZmCAMTA 1, 2, and 3 are dominantly expressed under heat stress response (El‐Sappah et al., 2022; Yue et al., 2015). Further, these TFs specifically bind to the heat shock elements (HSE) present in the promoter region of HSP genes and elicit their expression level (Zhang et al., 2020b). The EF‐Tu protein synthesis elongation factors are known for imparting heat stress tolerance in crops (Bukovnic et al., 2009; Fu et al., 2012; Ristic et al., 2008) including maize (Momčilović & Ristic, 2007). Besides the CaM‐mediated pathway, CNGCs and glutamate heat receptor‐like channels also transfer stress signals through Ca^2+^‐dependent protein kinases activation, along with calcineurin B‐like protein, and its interacting protein kinase (CIPK) and phosphoinositide‐specific phospholipase C (Chen et al., 2011; Hashimoto & Kudla, 2011; Reddy et al., 2004; Zhao et al., 2021). Phosphoinositide‐specific phospholipase C hydrolyze membrane‐bound phosphatidylinositol‐4,5‐bisphosphate (PIP_2_) into (IP_3_) and diacylglycerol after being activated by G‐protein coupled receptor (GPCR). This IP_3_ helps in the release of Ca^2+^ from the endoplasmic reticulum and ultimately joins the Ca^2+^‐mediated signaling cascade (Horváth et al., 2012; Ruelland et al., 2002). The mostly reported PLCs having a role in heat stress tolerance are PLC_3_, PLC_9_ (Hayes et al., 2021; Rupwate & Rajasekharan, 2012), and ZmPLC1 (in maize) (Zhai et al., 2013). At the end of the cascade, the activated CDPKs phosphorylate HSFs, which are imported into the nucleus for the expression of HSPs such as HSP40, HSP70, HSP90, and HSP100 (Qian et al., 2019) (Figure 3).

HSFs are grouped into three classes, namely, classes A, B, and C. Some of the class A and class B HSFs identified for their role in heat stress tolerance are ZmHsf1, 4, 5, 6, 17 (class A) and ZmHsf3, 11, 25 (class B) among the total 31 HSFs reported in maize (Lin et al., 2011; Zhang et al., 2020a). However, only class A HSFs have transcription activation domain (Kotak et al., 2004). It has been documented that HSFA1 is the master transcription activator, and it is sequestered in the cytoplasm by binding with chaperons (HSP70 and HSP90) and co‐chaperones during normal condition (Ohama et al., 2017). Conversely, the chaperones are recruited away from the HSFA1 during heat stress. Subsequently, HSFA1 trimerizes and transported into the nucleus for the activation of heat stress responsive genes (Yan et al., 2020; Zhao et al., 2021). A parallel signaling cascade initiate with the production of ROS production under heat stress (Hasanuzzaman et al., 2012, 2013). Membrane‐bound NADPH oxidase, which belongs to the respiratory burst oxidase homolog D family produces superoxide radical (O_2_ **^−^**) from molecular oxygen (O_2_). The half‐life of O_2_ **^−^** is extremely short, and it is dismutated to a more stable ROS species hydrogen peroxide (H_2_O_2_) either spontaneously or by the apoplastic superoxide dismutase (SOD) enzyme (Bahuguna & Jagadish, 2015). Eventually, H_2_O_2_ enters into the cytoplasm to activate some HSFs either directly or indirectly. In maize, H_2_O_2_ activates the MAPKs, specifically MAPK3 and MAPK6, which in turn activate CDPKs. This is followed by phosphorylation of HSFs that ultimately induce the expression of HSPs (Luna et al., 2011; Matika & Loake, 2014). On the other hand, H_2_O_2_ also directly activates HSFA1α, HSFA4α, and HSFA8 along with nitric oxide (NO) signaling to induce heat response gene expression in maize (El‐Sappah et al., 2022) (Figure 3).

Besides, upstream signaling initiating at the plasma membrane, a specific unfolded protein responses takes place in ER when the concentration of misfolded proteins increases rapidly under heat stress (Neill et al., 2019; Vitale & Boston, 2008). Misfolded proteins induce the recruitment of binding immunoglobulin protein (BIP), which dissociates from a non‐competent, monomeric form of inositol requiring enzyme‐1 (IRE1) (Carrara et al., 2015). BIPs help misfolded proteins to bind with IRE1 and cause dimerization. Dimeric IRE1 acts as dual ribonuclease and kinase to splice basic leucine zipper domain (ZmbZIP60) mRNA, so that it can code for ZmbZIP60 TF specific to heat stress after being imported into the nucleus (Reimold et al., 2000). Another way of activating UPR in ER is through the proteolytic cleavage of ER‐membrane protein, ZmbZIP17, and ZmbZIP28 in Golgi, followed by transport of cleaved globular domains, which act as TFs, into the nucleus, and activates the expression of HSPs (Jia et al., 2009; Zhang et al., 2017). Besides ROS, Ca^2+^ and other secondary messengers, phytohormones such as indole acetic acid, cytokinin, gibberellic acid, abscisic acid, salicylic acid, ethylene, brassinosteroids, and jasmonic acid are also documented for their role in signal transduction pathways during heat stress tolerance in plants (Eyidogan et al., 2012; Li et al., 2021). Huang et al. (2016) reported that the application of ABA along with the expression of AREB1/ABF1 TF showed enhanced thermotolerance in maize (Figure 3).

Although the response of maize crop under heat stress is relatively well characterized, it has been observed that maize plants exposed to long‐term heat stress may be subjected to physiological drought stress particularly under rainfed condition (Seetharam et al., 2021). Moreover, heat and drought stress in combination is an inevitable consequence under hot and dry environments, and more frequent under changing climate (Haddad et al., 2022). However, there are limited studies reporting gene(s) exclusively expressed under heat and drought stress combination. Qin et al. (2007) reported that maize plants overexpressed with ZmDREB2A (ZmDREBA2‐OE) showed drought tolerance by activating the expression of late embryonic abundant genes. This TF also helped in the upregulation of detoxification and thermotolerance related genes, and among those four genes including At5G03720 were upregulated by seven folds under heat stress. Similarly, *OsERF115/AP2EREBP110* gene, which belongs to the AP2/EREBP TF family has been identified in rice, which has an important role in both heat and drought tolerance (Suzuki et al., 2016). Thus, the identification of genes, which are exclusively expressed under heat and drought combination are crucial to understand maize response to a complex environment having stress interactions at spatial and temporal scales.

## 6 FUTURE PERSPECTIVES

Besides the impact of gradually increasing global mean temperature on weather patterns, crop phenology, and ecosystem services, incidences of heat stress at regional levels can cause severe yield losses in major cereal crops including maize. Advancement in phenomics, and other omics technologies, molecular markers, and genome editing tools provide new avenues for crop improvement programs. There are potential areas to prioritize for gaining a better understanding for heat stress tolerance in maize and develop promising thermotolerant maize genotypes. Some of these include the following:

- With limiting water resources, frequent co‐occurrence of heat and drought events are inevitable, which could be devastating to maize production when they coincide at sensitive reproductive and grain filling phase. Crop improvement programs for climate resilience are amongst the top priority for adaptation to climate change. However, identifying potential traits, QTLs, and genes that can be introgressed in the elite cultivars is a major challenge to expedite the development of climate‐resilient genotypes. Most of the studies in the past have been primarily focused on the single stress, which achieved limited success in long‐term performance of crops in the field conditions where stress interactions in a single crop cycle are very common. Indeed, several stress combinations have been suggested, which are very obvious in the field conditions (Mittler, 2006; Mittler & Blumward, 2010; Suzuki et al., 2014). Therefore, crop improvement programs should prioritize the focus on novel traits, QTLs, and genes, which are effective under single as well as a combination of two or more stresses.
- In the United States, the highest agricultural loss of $200 billion had been reported only due to the combination of heat waves and drought stress during 1980–2012 (http://www.ncdc.noaa.gov/billions/events). Despite of the relevance of studying plant responses under heat and drought combination stresses, there is a lack of experimental programs, dealing with combination stresses particularly heat and drought stress together. Mittler (2006), and Anwar et al. (2021) explained that a combination of stresses induces a unique set of genes, proteins, and metabolites, which are not expressed under individual stress conditions. Identification of traits and potential donors to develop a mapping population would help in understanding crop response to individual and combination of stresses. Moreover, different traits sensitive to high day, high night temperature and the interaction of temperature with other environmental factors (e.g., VPD and photoperiod) and edaphic factors (e.g., salt and nutrient stress) need to be explored in order to understand the physiological, biochemical, and molecular basis of heat tolerance in maize.
- Pollen viability and other characteristics of pollen growth are severely affected by heat stress in many crops. While male reproductive organs particularly are more susceptible to heat stress as compared to the female counterparts in some crop species such as sorghum (Djanaguiraman et al., 2018), pearl millet pistil was reported to be relatively more sensitive to heat stress (Djanaguiraman et al., 2018). Therefore, a better understanding of the relative tolerance levels of pollen and ovule in maize and associated metabolic and biochemical changes would need further studies to determine mechanistic understanding of reproductive stage heat stress tolerance in maize.
- Jiang et al. (2019) explained that secondary metabolites, that is, flavonoids particularly flavonols have an important role in thermotolerance by scavenging ROS in pollen and leaves in tomatoes. Conversely, Li et al. (2021) reported that key genes regulating flavonoid biosynthesis are highly expressed in drought overly insensitive mutants of maize seedlings under drought stress. Indeed, flavonoids are found as important metabolites accumulating under heat and salinity combination stress in rice (Jan et al., 2021). Therefore, the role of flavonoids and other secondary metabolites under heat stress and combination stresses may be explored in detail to understand their role in maize under heat stress.
- Under moderate heat stress conditions, it was determined that a significant increase in respiration, rather than relative decrease in photosynthesis, impacted yield loss in rice (Li et al., 2021). Therefore, a better understanding of the carbon‐balance dynamics that is optimizing carbon and energy use under stress would be critical to determine the biomass and yield. Hence, further studies are warranted to explore the role of respiratory carbon losses particularly during grain filling stage in maize.

## 7 CONCLUSIONS

Heat stress tolerance is an important trait to increase and maintain crop yield under current and future climate conditions. Screening of wild accessions, landraces, and mutant plant population to identify promising trait(s) and donors could be critical for breeding approaches to improve heat stress tolerance in crops. Moreover, mining and mapping of QTL(s) with the help of advanced molecular markers could be utilized to identify major/minor gene(s) contributing to heat stress tolerance. However, the majority of the molecular mechanistic studies with narrow genetic background conducted under controlled condition are not enough to elucidate plants’ response under natural condition. In addition, numerous challenges exist including lack of experimental facilities at the field levels to conduct heat stress studies under natural environment. Conversely, uncertainty of the occurrence of heat stress at the particular stage with desired intensity is also obvious, which requires precise experimental designing. Hence, low‐cost phenotyping facilities that can help in characterizing genotypes by manipulating air temperature under natural condition are required. Nevertheless, the complex interaction between high temperature and other climatic factors particularly drought and VPD need in‐depth understanding. Stresses in combination have become more prevalent under climate change. Hence, novel trait identification should be prioritized for stress combinations, where plant responses under combined stresses could be unique and different when compared to single stress under isolated environments. Besides, traditional and new genetic tools, robust and efficient phenotypic tools, for the acquisition of bulk and reproducible phenotypic data should be utilized in targeted crop improvement programs to enhance heat stress tolerance in maize and other food grain crops. Hence, such studies deserve enough experimental fundings for the interest of climate‐resilient crop improvement.

## AUTHOR CONTRIBUTIONS

**Ivica Djalovic**: Conceptualization, Investigation, Methodology, Project administration, Validation, Writing‐original draft, Writing‐review & editing. **Sayanta Kundu**: Conceptualization, Data curation, Investigation, Methodology, Writing‐original draft, Writing‐review & editing. **Rajeev Nayan Bahuguna**: Conceptualization, Data curation, Investigation, Writing‐original draft, Writing‐review & editing. **Ashwani Pareek**: Conceptualization, Validation, Writing‐original draft, Writing‐review & editing. **Ali Raza**: Validation, Writing‐original draft, Writing‐review & editing. **Sneh L. Singla‐Pareek**: Conceptualization, Methodology, Validation, Writing‐original draft, Writing‐review & editing. **P. V. Vara Prasad**: Conceptualization, Funding acquisition, Investigation, Project administration, Supervision, Writing‐original draft, Writing‐review & editing. **Rajeev K. Varshney**: Validation, Writing‐original draft, Writing‐review & editing.

## CONFLICT OF INTEREST STATEMENT

The authors declare no conflicts of interest.

## Data Availability

Data availability or sharing is not applicable to this review article as no data were generated.
